# Complete Chloroplast Genomes and Comparative Analysis of Sequences Evolution among Seven *Aristolochia* (Aristolochiaceae) Medicinal Species

**DOI:** 10.3390/ijms20051045

**Published:** 2019-02-28

**Authors:** Xiaoqin Li, Yunjuan Zuo, Xinxin Zhu, Shuai Liao, Jinshuang Ma

**Affiliations:** 1 Shanghai Chenshan Plant Science Research Center, Chinese Academy of Sciences, Shanghai Chenshan Botanical Garden, Shanghai 201602, China; lxq@sibs.ac.cn (X.L.); zuo.yunjuan@gmail.com (Y.Z.); 2 Shanghai Center for Plant Stress Biology, Chinese Academy of Sciences, Shanghai 201602, China; 3 University of Chinese Academy of Sciences, Shanghai 201602, China; 4 College of Life Sciences, Xinyang Normal University, Xinyang 464000, Henan, China; huamixinhua1@gmail.com; 5 School of Life Sciences, East China Normal University, Shanghai 200241, China; shuai_liao@bjfu.edu.cn

**Keywords:** *Aristolochia*, chloroplast genome, molecular evolution, compare analysis, phylogeny

## Abstract

Aristolochiaceae, comprising about 600 species, is a unique plant family containing aristolochic acids (AAs). In this study, we sequenced seven species of *Aristolochia*, and retrieved eleven chloroplast (cp) genomes published for comparative genomics analysis and phylogenetic constructions. The results show that the cp genomes had a typical quadripartite structure with conserved genome arrangement and moderate divergence. The cp genomes range from 159,308 bp to 160,520 bp in length and have a similar GC content of 38.5%–38.9%. A total number of 113 genes were identified, including 79 protein-coding genes, 30 tRNAs and four rRNAs. Although genomic structure and size were highly conserved, the IR-SC boundary regions were variable between these seven cp genomes. The *trnH*-GUG genes, are one of major differences between the plastomes of the two subgenera *Siphisia* and *Aristolochia*. We analyzed the features of nucleotide substitutions, distribution of repeat sequences and simple sequences repeats (SSRs), positive selections in the cp genomes, and identified 16 hotspot regions for genomes divergence that could be utilized as potential markers for phylogeny reconstruction. Phylogenetic relationships of the family Aristolochiaceae inferred from the 18 cp genome sequences were consistent and robust, using maximum parsimony (MP), maximum likelihood (ML), and Bayesian analysis (BI) methods.

## 1. Introduction

*Aristolochia* sensu lato, comprising about 500 species, is the largest genus of Aristolochiaceae, with a broad distribution range from tropical to subtropical, extending to temperate regions [1,2]. Several species of *Aristolochia*, such as *Aristolochia moupinensis*, *Aristolochia tagala*, and *Aristolochia mollissima*, have been reported as traditional Chinese medicines [3,4]. Aristolochiaceae is a unique plant family containing aristolochic acids (AAs), and their derivatives are widely implicated in liver cancers [5,6]. However, current studies have demonstrated that AAs are of nephrotoxicity, carcinogenicity, and mutagenicity [7,8]. The sale and use of AA-containing herbal preparations have been restricted in many countries [9].

The monophyly of Aristolochiaceae was well supported in most analysis, and was divided into two subfamilies, Asaroideae and Aristolochioideae [10,11]. The studies recognized two genera *Saruma* and *Asarum* in Asaroideae [10,11,12,13]. The genus *Aristolochia* of subfamily Aristolochioideae was classified into two major lineages, as indicated by previous studies based on morphological characters and molecular phylogenetic methods [10,14,15,16]. In the past years, the nuclear *ITS*2, *phyA* gene, and several plastid genome regions (such as *matk*, *rbcL*, *trnK*, and *trnL*-*trnF*) or their combinations have been frequently used in molecular systematics of Aristolochiaceae [11,15,17,18]. The inter-simple sequence repeat (ISSR) markers were also used to identify diverse genetic stocks and understand the evolutionary relationships of *Aristolochia* [19,20]. The selected loci failed to provide sufficient phylogenetic information to elucidate the evolutionary relationships among *Aristolochia* species. A universal barcode either using whole chloroplast (cp) genomes or hyper-variable regions are urgently needed, which may significantly improve the low resolution in plant relationships and contribute to the conservation, domestication, and utilization of *Aristolochia* plants.

The chloroplast is the key organelle for photosynthesis and carbon fixation in green plants [21]. Their genomes could provide valuable information for taxonomic classification and phylogenetic reconstruction among species of land plants [22,23,24,25]. Typical cp genomes in angiosperms have a generally conserved quadripartite circular structure with two copies of inverted repeat (IR) regions that are separated by a large single copy (LSC) region and a small single copy (SSC) region, and encode 120–135 genes with sizes in the range of 120–170 kb [26,27]. In recent years, the cp genomes of *Aristolochia debilis*, *Aristolochia contorta*, *Saruma henryi*, and nine species of *Asarum* within the Aristolochiaceae family have been reported [28,29,30,31]. Those sequenced cp genomes of Aristolochiaceae, except for those of *Asarum* species, were conserved in length, gene and GC content, from which no rearrangement event had been detected. 

With the rapid development of next-generation sequencing (NGS), it is now more convenient and cheaper to obtain cp genome sequences, feasible to compare analysis of sequences evolution among different individuals. In this study, we reported seven complete cp genomes of *Aristolochia* and conducted comparative genomic analyses, focused on gene size, content, patterns of nucleotide substitutions, and variable sites. Another 12 published cp genome sequences of Magnoliids downloaded from the National Center for Biotechnology Information (NCBI) organelle genome database (https://www.ncbi.nlm.nih.gov) [32] were also used to detect selective sites, repeat sequences, simple sequence repeats (SSRs), and phylogenetic constructions. We performed these comparative genomes analysis to obtain comprehensive understanding the structure of plastomes within *Aristolochia* and to provide genetic resources for future research in the genus.

## 2. Results

### 2.1. The Chloroplast Genome Structures of Species

**Figure 1:**
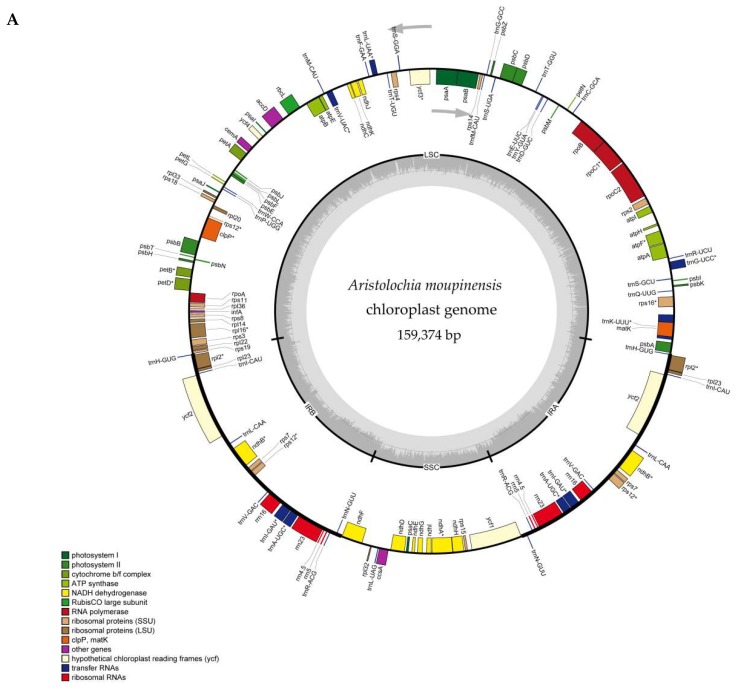

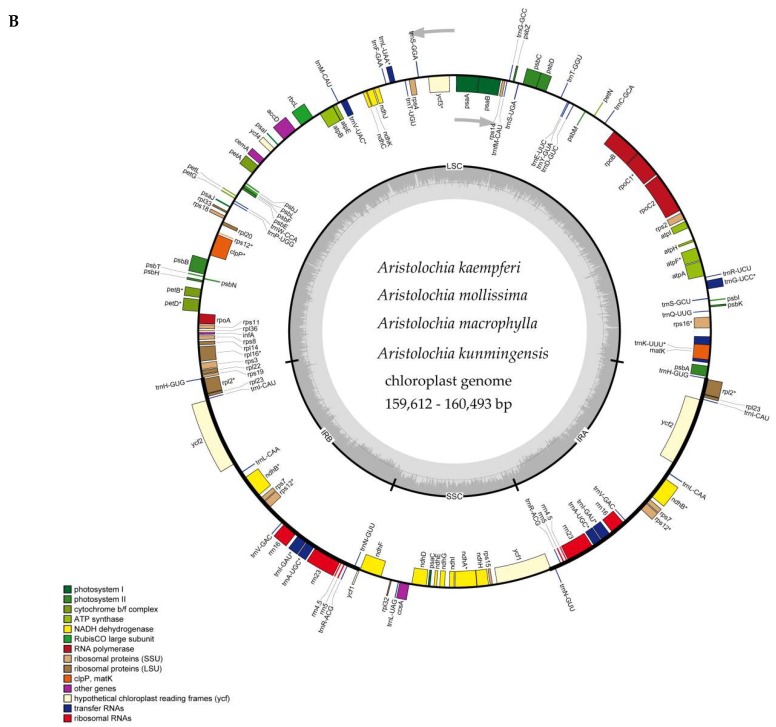

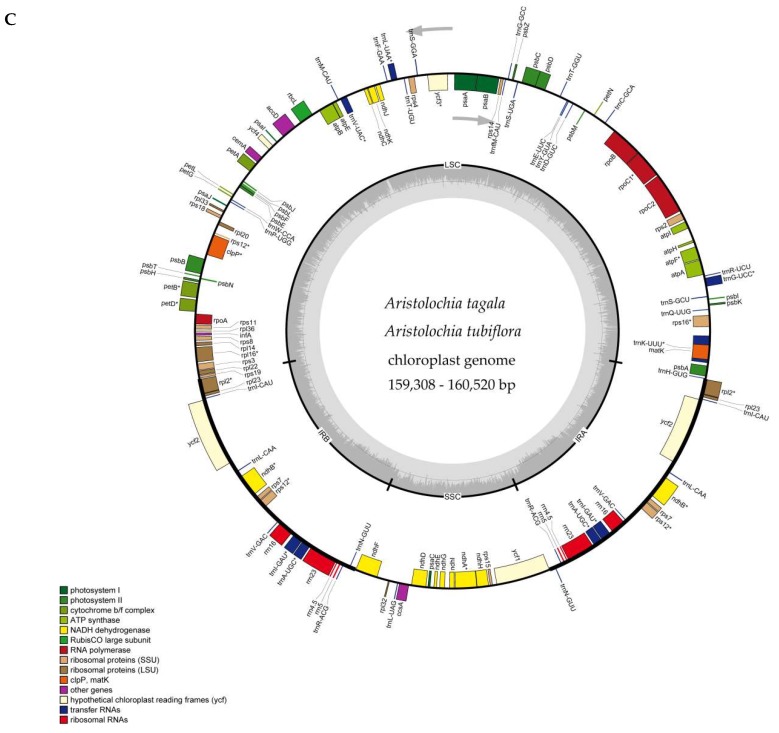
Gene maps of the complete cp genome of seven species of *Aristolochia*. Gene map of cp genome of (**A**) *Aristolochia manshuriensis*; (**B**) *Aristolochia kaempferi*, *Aristolochia macrophylla*, *Aristolochia mollissima* and *Aristolochia kunmingensis*; (**C**) *Aristolochia tagala* and *Aristolochia tubiflora*. Genes on the inside of the circle are transcribed clockwise, while those outside are transcribed counter clockwise. The darker gray in the inner circle corresponds to GC content, whereas the lighter gray corresponds to AT content.

**Figure 2:**
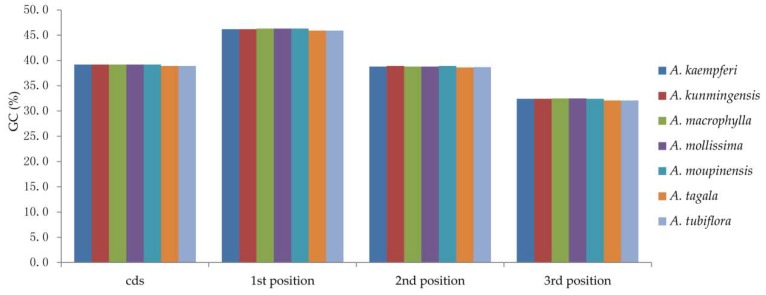
The GC (%) composition in different positions of coding sequence (CDS) region of species within *Aristolochia*.

All the species of *Aristolochia* we sequenced had a typical quadripartite structure, with a circular molecule of 159,308 bp to 160,520 bp in length. The complete cp genomes of involved species comprise an LSC region (88,652–89,859 bp) and an SSC region (19,322–19,799 bp), separated by a pair of IRs ranging from 25,242 bp to 25,700 bp in length (Figure 1, Table 1). GC content of the plastomes of the seven *Aristolochia* species varies slightly from 38.5% to 38.9% (Table 1). The GC content within coding sequence (CDS) of the two species (*A. tagala* and A. *tubiflora*) of subgenus *Aristolochia* and five species (*A. kunmingensis*, *A. moupinensis*, *A. macrophylla*, *A. kaempferi*, and *A. mollissima*) of subgenus *Siphisia* was 38.9% and 39.2%, respectively. GC% content of the first position was higher compared to those of the second and third positions (Figure 2, Table S1). A total of 113 unique genes were identified in the seven cp genomes, including 79 protein coding genes, 30 tRNAs and four rRNAs, 19 or 18 genes of which duplicated in the IR region (Table 1 and Table 2).

Introns play an important role in the regulation of some gene expressions [33]. Eighteen genes of seven plastomes contain one intron, including *atpF*, *rpoC1*, *ycf3*, *rps12*, *rpl2*, *rpl16*, *clpP*, *petB*, *petD*, *rps16*, *ndhA*, *ndhB*, and six tRNA genes, while three genes (*clpP*, *ycf3*, and *rps12*) contain two introns. The longest intron occurred in the *trnK*-UUU gene is 2552–2687 bp of seven plastomes, and had been used to the inter- and intra-species of *Aristolochia* [2,16]. In addition, the length of *rpl2* intron in species of subgenus *Siphisia* and subgenus *Aristolochia* is 700 bp and 659 bp, respectively (Table 3).

### 2.2. IR Contraction and Expansion

The IR regions are expanded in five species of subgenus *Siphisia* compare with other two species (*A. tagala* and *A. tubiflora*) of subgenus *Aristolochia*, indicated by different duplication genes in the IR regions, where eight or seven tRNA genes were duplicated, respectively (Figure 1, Table 2). The size of the IR region of subgenus *Siphisia* varies from 25,664 bp to 25,700 bp, and is 25,242 bp and 25,431 bp in the two plastomes of subgenus *Aristolochia* (Table 1).

**Figure 3:**
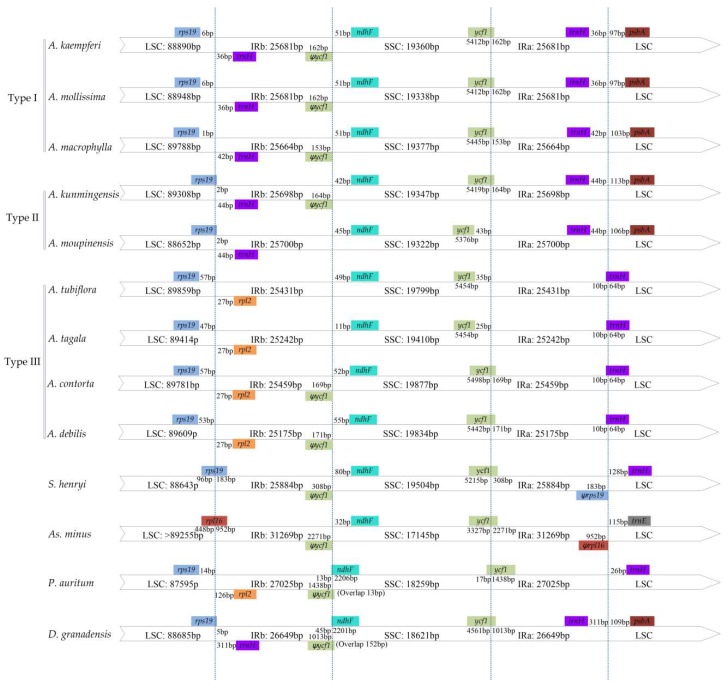
Comparison of the borders of large single copy (LSC), small single copy (SSC) and inverted repeat (IR) regions among 13 cp genomes. Number above the gene features means the distance between the ends of genes and the borders sites. These features are not to scale.

Fluctuation of IR-SC borders, together with the adjacent genes, were examined among seven *Aristolochia* species and six plastomes retrieved from GenBank (including *Aristolochia contorta*: NC_036152.1, *Aristolochia debilis*: NC_036153.1, *Asarum canadense*: MG544845-MG544851, *Saruma henryi*: MG520100, *Piper auritum*: NC_034697.1, and *Drimys granadensis*: NC_008456.1) (Figure 3). The LSC-IRb border, was located within the genic spacer of *rps19*-*trnH* for *A. kaempferi*, *A. macrophylla*, and *A. mollissima* (Type I), within the *rps19* gene for *A. kunmingensis* and *A. moupinensis* (Type II), while in the *rps19*-*rpl2* spacer for *A. tagala* and *A. tubiflora* (Type III). There were two types of SSC-IRa border among 13 detected species. In the three plastomes (*A. moupinensis*, *A. tubiflora*, and *A. tagala*), which *ycf1* gene was fully located in the SSC region, and 25-43 bp apart from the SSC-IRa border. The SSC-IRa border was situated in the coding region *ycf1* gene in the other 10 sequenced species, which spanned into the IRa region. Among the 10 detected species, the pseudogene *ycf1* in the IRb region with the same length as far as the IRa expanded into *ycf1* gene, and the length ranged from 153 bp to 2271 bp. The *ndhF* gene was entirely located in the SSC region in 10 species of Aristolochiaceae, but varied in distance (11-80 bp) from the IRb-SSC border. The LSC-IRa border in the species of subgenus *Aristolochia* was situated in the *trnH* gene with 10 bp into the IRa region (Type III), while the border was located in the *trnH*-*psbA* spacer in subgenus *Siphisia* species (Type I and II) (Figure 3).

### 2.3. Codon Usage

**Figure 4:**
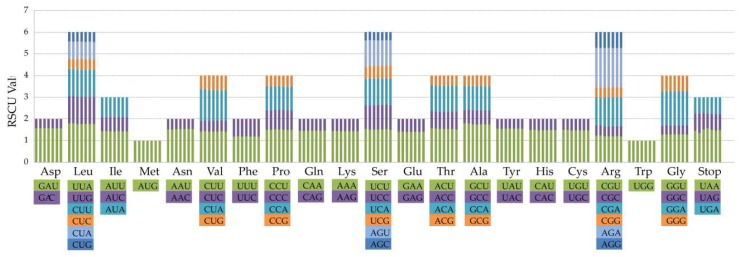
Codon content of 20 amino acid and stop codons in all protein-coding genes of the seven cp genomes. The histogram from the left-hand side of each amino acid shows codon usage within *Aristolochia* (From left to right: *A. tagala*, *A. tubiflora*, *A. moupinensis*, *A. kunmingensis*, *A. kaempferi*, *A. macrophylla*, and *A. mollissima*).

All the protein-coding genes were composed of 26,194–26,398 codons in the cp genomes of the seven species of *Aristolochia*. The codon usages of protein-coding genes in the cp genomes are summarized in Figure 4 and Table S2. Among these codons, the most common amino acid in the protein-coding genes is leucine, which appears 2775 times in *A. kaempferi* and *A. mollissima*. The relative synonymous codon usage (RSCU) value analysis showed that almost all amino acids have more than one synonymous codon, except methionine and tryptophan. Nearly all of the protein-coding genes of *Aristolochia* species had the standard ATG start codon (RSCU = 1). About half of codons have RSCU > 1, and most of those (29/31, 93.5%) end with base A or T. About half of the codons have RSCU < 1, and most of those (28/31, 90.3%) end with base C or G.

### 2.4. Positive Selection Analysis

We compared the ratio of non-synonymous (dN) and synonymous (dS) substitution for 79 protein-coding genes among seven species, including *A. kunmingensis*, *A. kaempferi*, *A. tagala*, *A. debilis*, *As. canadense*, *S. henryi*, and *P. auritum* within Piperales. The statistical neutrality test showed that five genes in the seven cp genomes are under significant positive selection, and these genes are involved in the synthesis of ribosomal small and large subunit protein (*rps12*, *rps18*, and *rpl20*) or unknown function (*ycf1* and *ycf2*) (Table 4). Likelihood ratio tests (M1a vs. M2a, M7 vs. M8) supported the presence of positively selected codon sites (*p* < 0.05) (Table S3). According to the M2a and M8 models, the *rpl20* harbored three or four sites under positive selection. The gene *ycf1* harbored one or three sites under positive selection based on two models, respectively. In addition, we identified *rps12* gene with one positive selection site.

### 2.5. Repeat Structure and Simple Sequence Repeats Analyses

**Figure 5:**
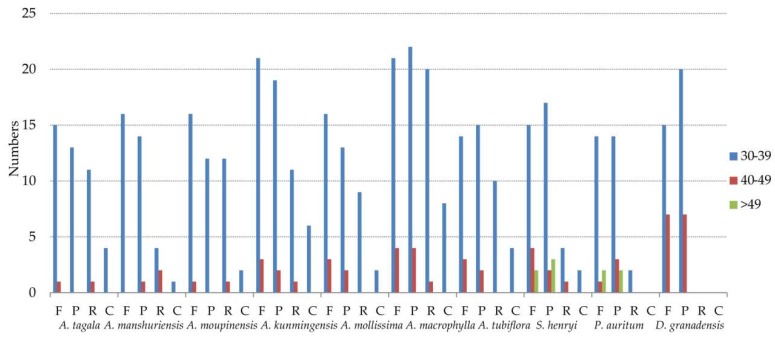
Repeat sequences in ten cp genomes. REPuter was used to identify repeat sequences with length ≥ 30 bp and sequence identity ≥ 90% in the cp genomes. F, P, R, and C indicate the repeat types F (forward), P (palindrome), R (reverse), and C (complement), respectively. Repeats with different lengths are indicated in different colors.

**Figure 6:**
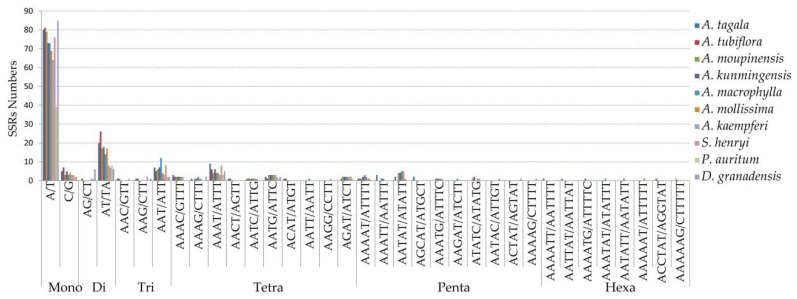
Frequency of simple sequence repeats (SSRs) in the ten cp genomes.

Repeats in ten cp genomes were analyzed using REPuter, including seven species of *Aristolochia*, *S. henryi*, *P. auritum*, and *D. granadensis* (Figure 5, Table S4). The results showed that *A. macrophylla* had the greatest number of repetitive elements in cp genome, comprised of 25 forward, 26 palindromic, 21 reverse, and eight complement repeats. The size of the most repeats were 30–39 bp, and the repeats with the length > 49 bp only occurred in cp genomes of *S. henryi* and *P. auritum*. The longest repeats, with a length of 1591 bp, was detected in *S. henryi*. The total numbers of SSRs were also identified in the cp genomes of the ten species (Figure 6 and Table S5). Mononucleotide repeats were the largest in a number of these SSRs, with 88% and 85% found in A. *tubiflora* and *A. tagala*, respectively. A/T repeats were the most common of mononucleotides, while AT/TA repeats are the majority of dinucleotide repeat sequences (96.3%–100%). The trinucleotide in the five species of subgenus *Siphisia* were only comprised of AAT/ATT repeats (100%), while *A. tubiflora* and *A. tagala* of subgenus *Aristolochia* also comprised AAC/GTT and AAG/CTT repeats.

### 2.6. Comparative Genomic Divergence and Hotspots Regions

**Figure 7:**
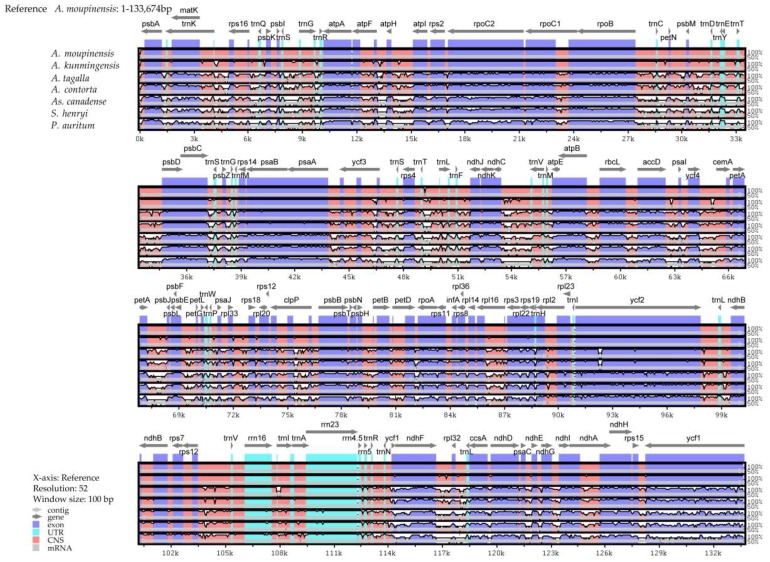
Sequence identity plot compared seven cp genomes with *A. moupinensis* as a reference by using mVISTA. Grey arrows and thick black lines above the alignment indicate genes with their orientation and the position of the IRs, respectively. A cut-off of 70% identity was used for the plots, and the *Y*-scale represents the percent identity from 50% to 100%.

The SC and IR regions of cp genomes of the seven species (including *A. moupinensis*, *A. kunmingensis*, *A. tagala*, *A. contorta*, *S. henryi*, *As. canadense*, and *P. auritum*) were compared using the mVISTA program to detect hyper-variable regions (Figure 7). The alignment revealed high sequence conservatism across the cp genomes of *A. moupinensis* and *A. kunmingensis* of subgenus *Siphisia*. The comparison among seven cp genomes showed that the IR region was more conserved than the SC regions. The most divergent regions located in the intergenic spacers, and the most divergent coding regions were *ndhF* and *ycf1*.

**Figure 8:**
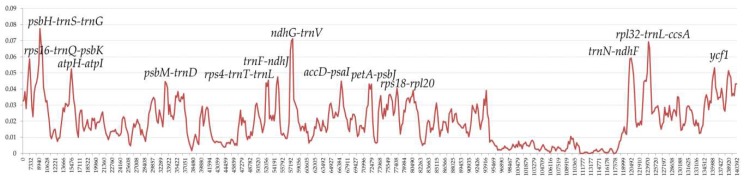
Sliding window analysis of the entire cp genome of seven *Aristolochia* species (window length: 600 bp; step size: 200 bp). *X*-axis: position of the midpoint of a window; *Y*-axis: nucleotide diversity of each window.

Comparative analysis among our seven sequenced species within *Aristolochia* was conducted of the entire cp genomes, LSC, SSC, IR, and CDS regions, respectively (Table 5). The nucleotide diversity (Pi) value was also calculated to evaluate the sequence divergence among these cp genomes, and their values varied from 0 to 0.07746 (Figure 8). The analysis revealed that the SSC region, compared with other regions, exhibited the highest levels of divergence (Pi = 0.03114). These values of the LSC region, varied from 0.00175 to 0.07746, with the mean value of 0.02182. The IR region exhibited the lowest Pi values varying from 0 to 0.01056, with the mean of 0.00411, indicating that IR region was the most conserved one. Furthermore, we identified 16 hotspot regions (Pi > 0.04, the mean value = 0.05413) with the full length of 20,296 bp, including *rps16*-*trnQ*-*psbK*, *psbI*-*trnS*-*trnG*, *atpH*-*atpI*, *psbM*-*trnD*, *rps4*-*trnT*-*trnL*, *trnF*-*ndhJ*, *ndhC*-*trnV*, *accD*-*psaI*, *petA*-*psbJ*, *rps18*-*rpl20*, *trnN*-*ndhF*, *rpl32*-*trnL*-*ccsA*, and four regions of *ycf1* coding gene (Table 6). Ten of these (*rps16*-*trnQ*-*psbK*, *psbI*-*trnS*-*trnG*, *atpH*-*atpI*, *psbM*-*trnD*, *rps4*-*trnT*-*trnL*, *trnF*-*ndhJ*, *ndhC*-*trnV*, *accD*-*psaI*, *petA*-*psbJ*, and *rps18*-*rpl20*) are located in the LSC, and six (*trnN*-*ndhF*, *rpl32*-*trnL*-*ccsA* and *ycf1*) in the SSC region, which could be utilized as potential markers for the phylogeny reconstruction and species identification of this subgenus in further studies.

### 2.7. Phylogenetic Analyses

**Figure 9:**
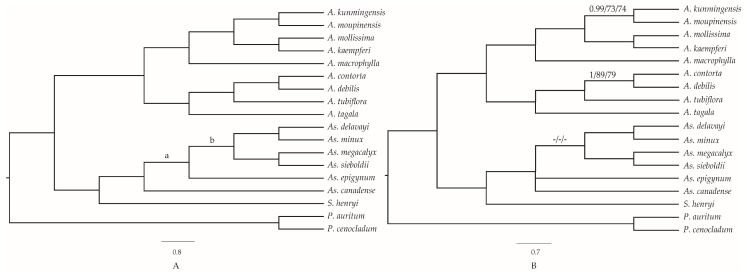
Phylogenetic relationships of the 18 species inferred from maximum parsimony (MP), maximum likelihood (ML), and Bayesian (BI) analyses. (**A**) The topology was constructed by cp genomes, LSC, SSC, CDS, and hotspots regions; (**B**) tree constructed by IR region. Bayesian posterior probability values < 0.95 or Bootstrap values < 90 were marked on the branches. The support values in node (a): 1/86/93 (using LSC region), 0.97/78/84 (SSC), 0.82/-/- (CDS), and 1/81/80 (hotspots); (b): 1/90/79 (SSC) and 1/73/71 (hotspots). Numbers above nodes are support values with Bayesian posterior probabilities values on the left, ML bootstrap values in the middle, and MP bootstrap values on the right. “ - “ indicates the value < 70.

The phylogenetic relationships of Aristolochiaceae were constructed based on six datasets (entire cp genome sequences except a copy of IR, LSC, SSC, IR, and CDS regions and combining 16 hotspots) of 18 samples, using three methods of ML, MP, and BI, respectively (Figure 9). The robust topologies were consistent for most clades of cp genomes, LSC, SSC, CDS, and hotspots datasets, with the high bootstrap values for most of the branches (Figure 9A). From these six different datasets, the phylogenetic analysis showed that the genera *Asarum* and *Saruma* represented by seven species formed a clade with posterior probabilities (PP) = 1 based on BI, bootstrap values (%) (BS) =100 based on ML and BS =100 based on MP methods. However, the tree constructed using sequences of the IR region failed to resolve the phylogeny position of *Asarum epigynum* and *As. canadense* (Figure 9B), maybe due to inadequate information sites in the IR region. These nine species of *Aristolochia* species formed another strongly supported monophyletic group (PP = 1; [ML] BS = 100; [MP] BS = 100), and were divided into two subclades with strong support, corresponding to the taxonomic division of subgenus *Siphisia* (PP = 1; [ML] BS = 100; [MP] BS = 100) and subgenus *Aristolochia* (PP = 1; [ML] BS = 100; [MP] BS = 100). Within the subgenus *Siphisia*, the species *A. macrophylla* from North America was sister to the rest of four species from Asian region (PP = 1; [ML] BS = 100; [MP] BS = 100).

## 3. Discussion

### 3.1. IR Contraction and Expansion

Taken another two reported species (*A. debilis* and *A. contorta*) of subgenus *Aristolochia* into account, although genomic structure and size were highly conserved, the IR-SC boundary regions were variable between these nine cp genomes of *Aristolochia* (Figure 3). In general, contraction and expansion at the borders of IR regions are common evolutionary events and may cause IR size variation of plastomes [29,34,35,36]. The length of the IR regions of five *Siphisia* species, varying in the range of 25,664–25,700 bp, was longer than those of the four species of subgenus *Aristolochia*, which varied from 25,175 bp to 25,459 bp (Table 1) [28]. We identified three types of the IR-SC junctions from the nine *Aristolochia* species, according to the organization of genes (Figure 3). Within five detected species of subgenus *Siphisia*, its patterns were Type I and II, while the Type III only occurred in the four species of subgenus *Aristolochia*. Type I was found in *A. mollissima*, *A. macrophylla*, and *A. kaempferi*, and was characterized by *trnH* gene in IR region and LSC-IRb border located in *rps19*-*trnH* spacer. Type II was only found in *A. moupinensis* and *A. kunmingensis* and refers to LSC-IRb border within the *rps19* gene. The *trnH* gene is intact and located upstream of *rpl2* in IRb region for type I and II. Type III pattern was found in the four species of subgenus *Aristolochia*, characterized by LSC-IRb and SSC-IRa border in the *rps19*-*rpl2* spacer and *trnH* gene, respectively. The *trnH* gene spanned the junction between IR-LSC regions in the four species of subgenus *Aristolochia*.

The shift of IR-LSC borders, caused by contraction and expansion of the gene *trnH*, is one of major differences between the plastomes of the subgenera *Siphisia* and *Aristolochia*. The whole gene duplication of *trnH* was detected in most monocots (e.g., *Acorus*, *Phalaenopsi* and *Dioscorea*), *D. granadensis* (Winteraceae) of magnoliids, and basal eudicots (*Ranunculus japonica* and *Ranunculus macranthus*) [34,37,38,39,40,41]. Wang et al. (2008) conducted RT-PCR assays and deduced that the duplicated *trnH* genes in most of non-monocots and monocots were regulated by different expression levels of promoters, and had distinct fates [37]. Within the family Aristolochiaceae, the *trnH* gene was located in the LSC region of *S. henryi*, 128 bp away from the border of LSC-IR, and was also a single copy in the six cp genomes of *Asarum*, but not sure the positions of the gene [29]. Furthermore, the study proposed that the low-complexity *trnH* region and ultimately inversion of a portion of the LSC were due to an AAT repeat. For inversion of a large portion of the LSC region, there were genes rearranged in SC-IR borders of sequenced species of *Asarum*, the IR boundaries of cp genomes of *Asarum* were highly variable and experienced positional shifts at borders. Such as there was an entirety of the SSC of *As. canadense* and *As. sieboldii* has been incorporated into the IR, and the boundary of the LSC-IR was found within *rpl2* or *rpl14* gene [29]. Within the species of *S. henryi*, *rps19* pseudogene existed in the IRa region, with the length of 183 bp. The *trnH*-*rps19* gene cluster had been used to distinguish monocots from other angiosperm for the organization of gene flanking the IR-SC junction [37,39]. The events of contraction or expansion of the IR regions also can be used to distinguish the species within Aristolochiaceae.

### 3.2. Inferring the Phylogeny and Species Identification of Aristolochia

Chloroplast genomes provide abundant resources significant for evolutionary, taxonomic, and phylogenetic studies [42,43,44]. The whole cp genomes and protein-coding genes have been successfully used to resolve phylogenetic relationships at multiple taxonomic levels during the past decade [45,46]. Repeats can lead to changes in genomic structure, and can be investigated to population genetics of allied taxa [47,48,49,50]. Repeats in ten cp genomes revealed that the repeats had a great number, comprised of 38–80 repeats (Figure 5 and Table S4), 66 and 138 repeats were respectively detected in *A. debilis* and *A. contorta* [28]. Given the variability of these repeats between lineages, they can be informative regions for developing genomic markers for phylogenetic analysis. SSRs, known as microsatellites, are tandemly repeated DNA sequences that consist of one–six nucleotide repeat units and are ubiquitous throughout the genomes [51]. A total number of 95–142 SSRs were identified in the seven cp genomes detected (Figure 6 and Table S5). According to the analysis of high variable regions, the hotspot regions within seven cp genomes also provide sufficient information sites to reveal phylogeny structure among species of family Aristolochiaceae, especially for the spacer *ycf1* and *rpl20*, with high nucleotide diversity and under positive selection (Table 4). The *ycf1* gene could be served as the barcode of land plants, and was also recognized as the most variable regions in plastid genome [50,52]. The gene *rpl20* is an important part of protein synthesis, and is involved in translation [53]. This study will also provide a reference for phylogenomic studies of closely related lineages among *Aristolochia* and other genera.

Furthermore, we can design effective markers for clarifying the phylogenetic relationships of *Aristolochia* and elucidating the evolutionary history of species complex of *Aristolochia* at the population level, based on the analysis of SSR and SNP sites. Understanding genetic variation within and between populations plays an important role in improving genetic diversity and is essential for future adaptive changes, reproduction patterns, and its conservation [20,54,55]. The cpDNA and B-class gene PISTILLATA (*PI*) have been used to investigate taxonomy at the species complex, such as *Aristolochia kaempferi* group, and these studies revealed that its DNA barcoding and taxonomy are difficult to assess for multiple hybridization and introgression events in the group [56,57]. More genes under selection and neutral markers should be used to clarify those multiple diversification events. It will better to apply the full genome information or hyper-variation regions to elucidate the species diversity of *Aristolochia*.

## 4. Materials and Methods

### 4.1. Plant Material, DNA Extraction, and Sequencing

We selected seven species according to their potential medicinal uses, including *A. kaempferi*, *A. kunmingensis*, *A. macrophylla*, *A. mollissima*, and *A. moupinensis* from subgenus *Siphisia* and *A. tagala* and *A. tubiflora* of subgenus *Aristolochia* (Table 7). Genomic DNA was isolated from silica-gel dried leaf tissue or herbarium specimens using Plant Genomic DNA Kit (TIANGEN, Beijing, China). DNA integrity was examined by electrophoresis in 1% (*w*/*v*) agarose gel and their concentration was measured using a NanoDrop spectrophotometer 2000 (Thermo Scientific; Waltham, MA, USA). The DNA was used to construct PE libraries with insert sizes of 150 bp and sequenced according to the manufacturer’s manual for the Illumina Hiseq X.

### 4.2. Chloroplast Genome Assembly and Annotation

We used the software Trimmomatic version 0.36 (Max Planck Institute of Molecular Plant Physiology, Potsdam, Germany) [58] to trim the low-quality reads. We retrieved the plastome sequence of *A. contorta* (NC_036152.1), *A. debilis* (NC_036153.1), *Asarum costatum* (AP018513.1), *Asarum minamitanianum* (AP018514.1), and *Asarum sakawanum* (AP017908.1) from GenBank and used these sequences as the references [28,30,31]. The plastome was assembled using mapping to reference genome and de novo methods as implemented in Geneious R11 (Biomatters, Auckland, New Zealand) [59].

The cp genomes of the seven species was annotated using the online program Dual Organellar GenoMe Annotator (DOGMA) (University of Texas at Austin, Austin, TX, USA) [60], Annotation of Organellar Genomes (GeSeq) [61] and Chloroplast Genome Annotation, Visualization, Analysis, and GenBank Submission (CPGAVAS) (Institute of Medicinal Plant Development, Chinese Academy of Medical Sciences and Peking Union Medical College, Beijing, China) [62]. The tRNA genes were confirmed using tRNAscan-SE software (v2.0, University of California, Santa Cruz, CA, USA) [63]. Plastome annotations were manually corrected with the software Artemis [64]. The gene map was drawn using the Organelle Genome DRAW (OGDRAW) [65,66] with default settings and checked manually. The complete cp genome sequences of the seven species were deposited in GenBank, accession numbers are MK503927-MG503933 (Table S6).

### 4.3. Genome Structure Analyses

The distribution of codon usage was investigated using the software CodonW (University of Texas, Houston, TX, USA) with the RSCU value [67]. GC content was analyzed using Molecular Evolutionary Genetics Analysis (MEGA v6.0, Tokyo Metropolitan University, Tokyo, Japan) [68]. REPuter program (https://bibiserv.cebitec.uni-bielefeld.de/reputer) (University of Bielefeld, Bielefeld, Germany) [69] was used to identify the size and location of repeat sequences, including forward, palindromic, reverse, and complement repeats in the seven cp genomes. For all repeat types, the minimal size was set as 30 bp and the two repeat copies had at least 90% similarity. Perl script MISA (https://webblast.ipk-gatersleben.de/misa/) [70] was used to detect microsatellites (mono-, di-, tri-, tetra-, penta-, hexanucleotide repeats) with the following thresholds (unit size, min repeats): ten repeat units for mononucleotide SSRs, five repeat units for dinucleotide SSRs, four repeat units for trinucleotide SSRs, and three repeat units each for tetra-, penta-, and hexanucleotide SSRs.

### 4.4. Positive Selection Analysis

To identify the genes under selection, we scanned the cp genomes of seven species within Piperales using codeml of the package PAML4 [71,72]. The software was used for calculating the non-synonymous (dN) and synonymous (dS) substitution rates, along with their ratios (ω = dN/dS). The analyses of selective pressures were conducted along the ML tree in Newick format (S7), which based on the whole CDS region was used to determine the phylogenetic relationships of these seven species. Each single-copy CDS sequences was aligned according to their amino acid sequence. We used the site-specific model with five site models (M0, M1a & M2a, M7 & M8) were employed to identify the signatures of adaptation across cp genomes. This model allowed the ω ratio to vary among sites, with a fixed ω ratio in all the branches. Comparing the site-specific model, M1a (nearly neutral) vs. M2a (positive selection) and M7 (β) vs. M8 (β & ω) were calculated in order to detect positive selection [73]. Likelihood ratio test (LRT) of the comparison (M1a vs. M2a and M7 vs. M8) was used respectively to evaluate of the selection strength and the p value of Chi square (χ²) smaller than 0.05 is thought as significant. The Bayes Empirical Bayes (BEB) inference [74] was implemented in site models M2a and M8 to estimate the posterior probabilities and positive selection pressures of the selected genes.

### 4.5. Genome Comparison and Nucleotide Variation Analysis

The whole-genome (minus a copy of IR region) alignment for the cp genomes of the seven species including our *A. moupinensis*, *A. kunmingensis*, A. *tubiflora* and four reported species (*A. contorta*, *As. canadense*, *S. henryi* and *P. cenocladum*) of Piperales, was performed and plotted by the mVISTA program (http://genome.lbl.gov/vista/mvista/submit.shtml) in Shuffle-LAGAN model [75,76], and with *A. moupinensis* as the reference. The seven cp genomes of *Aristolochia* were first aligned using MAFFT v7 [77] and then manually adjusted using BioEdit v7.0.9 [78]. Variable sites and nucleotide variability across complete cp genomes, LSC, IR, SSC, and CDS regions of seven species were calculated using DnaSP v5 [79]. Furthermore, for the seven cp genomes minus a copy IR region, a sliding window analysis was conducted to evaluate the nucleotide variability using DnaSP software. The step size was set to 200 base pairs, and the window length was set to 600 base pairs.

### 4.6. Phylogenetic Analyses

To estimate phylogenetic relationships within the Aristolochiaceae, plastomes of 18 taxa were compared, including nine samples from *Aristolochia*, six and one cp genomes from *Asarum* and *Saruma*, respectively (Table S5). A total of 11 cp genomes were downloaded from the NCBI database. In the phylogenetic analyses, *P. auritum* and *P. cenocladum* of *Piper* were used as outgroup. Phylogenetic trees were constructed by MP, ML and BI methods using the cp genomes, LSC, SSC, IR, CDS and hotspots regions. The sequences of the involved regions were aligned using MAFFT v7. MP analysis was performed with PAUP*4.0b10 [80], using a heuristic search performed 1000 replications and tree bisection-reconnection (TBR) branch swapping. BI was conducted using the program MrBayes v3.2 [81] with the GTR+I+G model at the CIPRES Science Gateway website (http://www.phylo.org/) [82]. The Markov Chain Monte Carlo (MCMC) analysis was run for 2,000,000 generations, sampling every 1000 generations. The posterior probabilities (PP) of the phylogeny and its branches were determined from the combined set of trees, discarding the first 25% trees of each run as burn-in, as determined by Tracer v1.7 [83]. Maximum likelihood phylogenies were constructed by a fast and effective stochastic algorithm using IQ-TREE v1.6.2 [84] with the Best-fit model by ModelFinder [85] according to Bayesian information criterion (BIC) and the robustness of the topology was estimated using 2000 bootstrap replicates. Figtree v1.4 (http://tree.bio.ed.ac.uk/software/figtree/) [86] was used to visualize and annotate trees.

## 5. Conclusions

The complete cp genomes of *A. kaempferi*, *A. kunmingensis*, *A. macrophylla*, *A. mollissima*, and *A. moupinensis* of the subgenus *Siphisia*, and *A. tagala* and *A. tubiflora* of the subgenus *Aristolochia* were reported in this study. The cp genomes length and gene content of the genus *Aristolochia* were comparatively conserved. Although genomic structure and size were highly conserved, the IR-SC boundary regions were variable between these nine cp genomes of *Aristolochia*. The whole duplicated *trnH* gene within five species of *Siphisia* is one of major differences between the plastomes of the subgenera *Siphisia* and *Aristolochia*. We also identified SSR sites, five positive selection sites and 16 variable regions, which provide a reference for developing tools to further study *Aristolochia* species. Furthermore, the phylogenetic constructions with six datasets of 18 cp genomes illustrated robust and consistent relationships with high supports.

## Figures and Tables

**Table 1:** Summary of complete chloroplast (cp) genomes of *Aristolochia* species.

Species	Total	LSC	IR	SSC	CDS	Total	Protein Coding Genes	tRNA	rRNA	GC%
*A. kaempferi*	159,612	88,890	25,681	19,360	79191.0	113	79	30	4	38.8
*A. kunmingensis*	160,051	89,308	25,698	19,347	79143.0	113	79	30	4	38.7
*A. macrophylla*	160,493	89,788	25,664	19,377	79116.0	113	79	30	4	38.6
*A. mollissima*	159,653	88,948	25,681	19,338	79194.0	113	79	30	4	38.8
*A. moupinensis*	159,374	88,652	25,700	19,322	78753.0	113	79	30	4	38.7
*A. tagala*	159,308	89,414	25,242	19,410	78582.0	113	79	30	4	38.5
*A. tubiflora*	160,520	89,859	25,431	19,799	78624.0	113	79	30	4	38.8

**Table 2:** Gene contents in the cp genomes of *Aristolochia* species.

No.	Group of Genes	Genes Names	Amount
1	Photosystems I	*psaA, psaB, psaC, psaI, psaJ*	5
2	Photosystems II	*psbA, psbB, psbC, psbE, psbF, psbH, psbI, psbJ, psbK, psbL, psbM, psbN, psbT, psbZ*	15
3	Cytochrome b/f complex	petA, petB *, petD *, petG, petL, petN	6
4	ATP synthase	atpA, atpB, atpE, atpF *, atpH, atpI	6
5	NADH dehydrogenase	ndhA *, ndhB *(x2), ndhC, ndhD, ndhE, ndhF, ndhG, ndhH, ndhI, ndhJ, ndhK	12 (1)
6	Rubisco large subunit	*rbcL*	1
7	RNA polymerase	rpoA, rpoB, rpoC1 *, rpoC2	4
8	Ribosomal proteins(SSU)	rps2, rps3, rps7(x2), rps8, rps11, rps12 **(x2), rps14, rps15, rps16 *, rps18, rps19	14 (2)
9	Ribosomal proteins(LSU)	rpl2 *(x2), rpl14, rpl16 *, rpl20, rpl22, rpl23(x2), rpl32, rpl33, rpl36	11 (2)
10	Assembly/stability of photosystem I	ycf3 **, ycf4	2
11	Transfer RNAs	*37/38 tRNAs (6 contain an intron, 7/8 in the IRs)*	37 (7)/38(8)
12	Ribosomal RNAs	*rrn4.5(x2), rrn5(x2), rrn16(x2), rrn23(x2)*	8 (8)
13	RNA processing	*matK*	1
14	Carbon metabolism	*cemA*	1
15	Cytochrome c synthesis	*ccsA*	1
16	Proteins of unknown function	*ycf1, ycf2(x2)*	3 (1)
17	Other genes	accD, clpP **, infA	3

* Gene contains one intron; ** gene contains two introns; (x2) indicates the number of the repeat unit is 2.

**Table 3:** Genes with introns in the seven cp genomes of *Aristolochia* as well as the lengths of the exons and introns.

Taxon	Gene	Location	Exon I	Intron I	Exon II	Intron II	Exon III
*A. kunmingensis*	*atpF*	LSC	144	792	411		
	*clpP*	LSC	71	912	288	674	250
	*ndhA*	SSC	551	1095	541		
	*ndhB*	IR	777	703	756		
	*petB*	LSC	6	215	642		
	*petD*	LSC	6	702	477		
	*rpl16*	LSC	9	1092	402		
	*rpl2*	IR	391	700	431		
	*rpoC1*	LSC	432	762	1617		
	*rps12*	LSC	114		232	536	23
	*rps16*	LSC	46	842	221		
	*trnA-UGC*	IR	38	804	35		
	*trnG-UCC*	LSC	24	763	48		
	*trnI-GAU*	IR	37	936	35		
	*trnK-UUU*	LSC	37	2574	35		
	*trnL-UAA*	LSC	35	454	50		
	*trnV-UAC*	LSC	37	587	36		
	*ycf3*	LSC	126	871	226	745	155
*A. mollissima*	*atpF*	LSC	144	772	411		
	*clpP*	LSC	71	897	295	672	243
	*ndhA*	SSC	551	1097	541		
	*ndhB*	IR	777	702	756		
	*petB*	LSC	6	215	642		
	*petD*	LSC	6	702	477		
	*rpl16*	LSC	9	1098	399		
	*rpl2*	IR	391	700	431		
	*rpoC1*	LSC	432	762	1617		
	*rps12*	LSC	114		232	536	23
	*rps16*	LSC	46	848	221		
	*trnA-UGC*	IR	38	804	35		
	*trnG-UCC*	LSC	24	764	47		
	*trnI-GAU*	IR	37	936	35		
	*trnK-UUU*	LSC	37	2562	35		
	*trnL-UAA*	LSC	35	455	50		
	*trnV-UAC*	LSC	37	587	36		
	*ycf3*	LSC	126	820	226	753	155
*A. kaempferi*	*atpF*	LSC	144	780	411		
	*clpP*	LSC	71	900	295	671	243
	*ndhA*	SSC	551	1097	541		
	*ndhB*	IR	777	702	756		
	*petB*	LSC	6	215	642		
	*petD*	LSC	6	702	477		
	*rpl16*	LSC	9	1101	399		
	*rpl2*	IR	391	700	431		
	*rpoC1*	LSC	432	765	1617		
	*rps12*	LSC	114		232	536	23
	*rps16*	LSC	46	842	221		
	*trnA-UGC*	IR	38	804	35		
	*trnG-UCC*	LSC	24	764	48		
	*trnI-GAU*	IR	37	936	35		
	*trnK-UUU*	LSC	37	2552	35		
	*trnL-UAA*	LSC	50	455	35		
	*trnV-UAC*	LSC	37	587	36		
	*ycf3*	LSC	126	812	226	752	155
*A. moupinensis*	*atpF*	LSC	144	789	411		
	*clpP*	LSC	71	909	288	669	250
	*ndhA*	SSC	551	1101	541		
	*ndhB*	IR	777	703	756		
	*petB*	LSC	6	211	646		
	*petD*	LSC	6	708	477		
	*rpl16*	LSC	399	1100	9		
	*rpl2*	IR	391	700	431		
	*rpoC1*	LSC	432	764	1617		
	*rps12*	LSC	114		232	536	23
	*rps16*	LSC	46	839	221		
	*trnA-UGC*	IR	38	804	35		
	*trnG-UCC*	LSC	24	758	48		
	*trnI-GAU*	IR	37	936	35		
	*trnK-UUU*	LSC	37	2567	35		
	*trnL-UAA*	LSC	35	462	50		
	*trnV-UAC*	LSC	37	587	36		
	*ycf3*	LSC	126	920	226	746	155
*A. macrophylla*	*atpF*	LSC	144	778	411		
	*clpP*	LSC	71	928	288	664	250
	*ndhA*	SSC	551	1084	541		
	*ndhB*	IR	777	702	756		
	*petB*	LSC	6	215	642		
	*petD*	LSC	6	706	477		
	*rpl16*	LSC	9	1095	402		
	*rpl2*	IR	391	700	431		
	*rpoC1*	LSC	432	788	1617		
	*rps12*	LSC	114		232	536	23
	*rps16*	LSC	46	836	221		
	*trnA-UGC*	IR	38	804	35		
	*trnG-UCC*	LSC	24	755	47		
	*trnI-GAU*	IR	37	936	35		
	*trnK-UUU*	LSC	37	2558	35		
	*trnL-UAA*	LSC	35	475	50		
	*trnV-UAC*	LSC	37	589	36		
	*ycf3*	LSC	126	892	226	757	155
*A.* *tubiflora*	*atpF*	LSC	144	751	411		
	*clpP*	LSC	71	819	288	671	250
	*ndhA*	SSC	551	1079	541		
	*ndhB*	IR	777	705	756		
	*petB*	LSC	6	214	642		
	*petD*	LSC	6	693	477		
	*rpl16*	LSC	9	1077	399		
	*rpl2*	IR	391	657	431		
	*rpoC1*	LSC	432	780	1617		
	*rps12*	LSC	114		232	536	23
	*rps16*	LSC	46	889	221		
	*trnA-UGC*	IR	38	809	35		
	*trnG-UCC*	LSC	24	768	47		
	*trnI-GAU*	IR	37	937	35		
	*trnK-UUU*	LSC	37	2635	35		
	*trnL-UAA*	LSC	35	514	50		
	*trnV-UAC*	LSC	37	594	36		
	*ycf3*	LSC	126	764	226	752	149
*A. tagala*	*atpF*	LSC	144	778	408		
	*clpP*	LSC	71	802	288	671	250
	*ndhA*	SSC	551	1101	541		
	*ndhB*	IR	777	704	756		
	*petB*	LSC	6	219	642		
	*petD*	LSC	6	488	477		
	*rpl16*	LSC	9	1071	399		
	*rpl2*	IR	391	657	431		
	*rpoC1*	LSC	432	785	1617		
	*rps12*	LSC	114		232	536	23
	*rps16*	LSC	46	848	221		
	*trnA-UGC*	IR	38	804	35		
	*trnG-UCC*	LSC	24	768	48		
	*trnI-GAU*	IR	37	743	35		
	*trnK-UUU*	LSC	37	2687	35		
	*trnL-UAA*	LSC	35	490	50		
	*trnV-UAC*	LSC	37	595	36		
	*ycf3*	LSC	126	830	226	763	149

**Table 4:** Positive selected sites detected in the cp genome of the Piperales.

Gene name	M2a		M8	
	selected sites	Pr (w > 1)	selected sites	Pr (w > 1)
*rpl20*	71A	0.918	71A	0.967 *
	72L	0.999 **	72L	1.000 **
	105R	0.963 *	105R	0.984 *
	116H	0.963 *	116H	0.988 *
*rps12*	79M	0.966 *	79M	0.987 *
*rps18*	4S	0.937	4S	0.975 *
	99T	0.921	99T	0.967 *
*ycf1*	206S	0.914	206S	0.967 *
	211V	0.975 *	211V	0.989 *
	1412N	0.922	1412N	0.971 *
*ycf2*	2036W	0.932	2036W	0.950 *

* *p* < 0.05; ** *p* < 0.01.

**Table 5:** Variable sites analyses in the seven *Aristolochia* cp genomes.

Regions	Number of Sites	Variable Sites	Parsimony Informative Sites	Nucleotide Diversity
LSC	94,564	4430	2315	0.02182
SSC	20,451	1433	804	0.03114
IR	25,884	253	154	0.00411
Complete	166,113	6422	3461	0.01717
CDS	79,365	2528	1376	0.01337

**Table 6:** Sixteen regions of highly variable sequences (Pi > 0.04) of *Aristolochia*.

High Variable Marker	Length	Variable Sites	Parsimony Informative Sites	Nucleotide Diversity
*rps16-trnQ-psbK*	1301	104	58	0.04278
*psbI-trnS-trnG*	2357	257	159	0.05364
*atpH-atpI*	1160	104	69	0.04439
*psbM-trnD*	1119	152	75	0.05888
*rps4-trnT-trnL*	1572	105	50	0.04178
*trnF-ndhJ*	920	85	51	0.04216
*ndhC-trnV*	1402	152	97	0.06311
*accD-psaI*	637	61	35	0.04220
*petA-psbJ*	1130	106	65	0.04444
*rps18-rpl20*	682	58	36	0.04155
*trnN-ndhF*	1492	161	86	0.04758
*rpl32-trnL-ccsA*	2679	202	113	0.04608
*ycf1a*	1225	126	74	0.04285
*ycf1b*	652	56	32	0.04053
*ycf1c*	1228	134	84	0.04611
*ycf1d*	740	70	39	0.04278
Combine	20296	2216	1349	0.05413

**Table 7:** Sampled species and their voucher specimens used in this study.

Species	Samples	Voucher	locality
*A. kaempferi*	E2265	Yuan Wang	Japan, Tokyo
*A. kunmingensis*	E754	Zhanghua Wang	China, Yunnan
*A. macrophylla*	E2111	Jinshuang Ma	North America, North Carolia
*A. mollissima*	E1016	Xinxin Zhu & Zhixiang Hua	China, Guangdong
*A. moupinensis*	E1086	Xinxin Zhu & Zhixiang Hua	China, Sichuan
*A. tagala*	E1071	Yuan Wang	China, Hongkong
*A. tubiflora*	E2239	Shuwan Li	China, Guangxi

## Supplementary Materials

Supplementary materials can be found at https://www.mdpi.com/1422-0067/20/5/1045/s1.

## Abbreviations

RSCU	Relative synonymous codon usage
NGS	Next-generation sequencing
TLA	Three letter acronym
SSR	Simple sequence repeats
ATP	Adenosine triphosphate
MP	Maximum parsimony
ML	Maximum likelihood
Pi	Nucleotide diversity
BI	Bayesian Inference
AAs	Aristolochic acids
LSC	Large single copy
SSC	Small single copy
CDS	Coding sequence
IR	Inverted repeat
SC	Single copy
CP	Chloroplast
